# Modified Logistic Regression Models Using Gene Coexpression and Clinical Features to Predict Prostate Cancer Progression

**DOI:** 10.1155/2013/917502

**Published:** 2013-12-04

**Authors:** Hongya Zhao, Christopher J. Logothetis, Ivan P. Gorlov, Jia Zeng, Jianguo Dai

**Affiliations:** ^1^Industrial Center, Shenzhen Polytechnic, Shenzhen, Guangdong 518055, China; ^2^Department of Genitourinary Medical Oncology, Unit 1374, The University of Texas MD Anderson Cancer Center, 1515 Holcombe Boulevard, Houston, TX 77030-4009, USA; ^3^School of Computer Science and Technology, Soochow University, Suzhou 215006, China; ^4^School of Applied Chemistry and Biotechnology, Shenzhen Polytechnic, Shenzhen, Guangdong 518055, China

## Abstract

Predicting disease progression is one of the most challenging problems in prostate cancer research. Adding gene expression data to prediction models that are based on clinical features has been proposed to improve accuracy. In the current study, we applied a logistic regression (LR) model combining clinical features and gene co-expression data to improve the accuracy of the prediction of prostate cancer progression. The top-scoring pair (TSP) method was used to select genes for the model. The proposed models not only preserved the basic properties of the TSP algorithm but also incorporated the clinical features into the prognostic models. Based on the statistical inference with the iterative cross validation, we demonstrated that prediction LR models that included genes selected by the TSP method provided better predictions of prostate cancer progression than those using clinical variables only and/or those that included genes selected by the one-gene-at-a-time approach. Thus, we conclude that TSP selection is a useful tool for feature (and/or gene) selection to use in prognostic models and our model also provides an alternative for predicting prostate cancer progression.

## 1. Introduction

Prostate cancer (PCa) is the second leading cause of cancer-related deaths among men in the USA [1, 2]. Screening using serum prostate-specific antigen (PSA) has improved the early detection of PCa and has resulted in an increase in the proportion of patients with disease that is curable by prostatectomy [3, 4]. However, 20% to 30% of treated patients will develop a local or metastatic recurrence which reflects the most adverse clinical outcome [4]. Thus, from the clinical perspective, it is important to be able to predict which patients will experience a relapse.

Traditional PCa prognosis models are based on some clinical features, such as pretreatment PSA levels, biopsy Gleason score (GS), and clinical stage, but in practice, they are inadequate to accurately predict disease progression [5]. With the development of microarray technology in recent years, a number of studies have been conducted to characterize the dynamics of gene expression in PCa progression by using DNA microarrays. In some studies, tumor expression signatures associated with clinical parameters and outcomes have been identified [6–9]. As a result, it is possible to develop the clinical models with the variables of gene signatures identified from microarray data and some clinical features to predict which men would experience progression to the metastatic form of PCa.

However, it has been found that none of the predictive models using gene expression profiles are significantly better than models using clinical variables only in predicting PCa progression [10, 11]. In fact, only a limited number of genes are used to avoid overfitting in these models. The genes are usually selected through a gene-by-gene comparison. The results of recent studies, however, suggest that assessing the expression of more than one gene (i.e., coexpression analysis) yields a better prediction of tumor progression than the analysis of individual genes does [12–15].

In this study, we tried to propose such models by merging the coexpressed genes' profiles and some clinical features to predict the patients who would suffer from PCa progression. The genes used in our models are identified by a top-scoring pair (TSP) algorithm. The TSP method was initially introduced by Geman et al. as a classification technique for microarray data [16]. We applied the TSP-based LR model to published microarray experiments whose patients suffered from PCa progression. We analyzed the effects of the number of coexpressed genes included in the models and the selection of the clinical variables on the accuracy of the prediction. We also compared the performance of the most commonly used classification methods with our proposed method.

## 2. Materials and Methods

### 2.1. Logistic Regression Model for the Classification of Gene Microarrays

Genome-wide microarray data from different cells give insight into the gene expression variation of various genotypes and phenotypes. Classification of patients is an important aspect of cancer diagnosis and treatment. For example, microarray experiments can be employed to screen gene expression levels from cancerous and normal phenotypes so that proper prediction rules can be built from these gene expression data. In this section, we introduce a logistic regression (LR) model to classify the phenotypes of microarray data.

We denote a gene expression matrix by *D* = {*x*_*ij*_}_*M*×*N*_, where there are *M* genes and *N* samples, and *x*_*ij*_ denotes the expression value of the *i*th gene, *i* ∈ {1,…, *M*}, from the *j*th sample, *j* ∈ {1,…, *N*}. The vector *G*_*i*·_ = (*x*_*i*1_,…, *x*_*iN*_) represents the *i* gene expression values over all *N* samples and *S*_·*j*_ = (*x*_1*j*_,…, *x*_*Mj*_) is the expression profile of all *M* genes for the *j*th sample. Let *y*_*j*_ be the binary phenotype of the *j*th profile: *y*_*j*_ = 0 indicates that the *j*th sample belongs to class 0 (e.g., normal tissues) and *y*_*j*_ = 1 indicates that the *j*th sample belongs to class 1 (e.g., tumor tissues).

The classification of microarray data has been intensively researched for years. But some limitations have stood out, such as the small-sample dilemma, “black box,” and lack of prediction strength [16–18]. We used LR to build the prediction models for a binary outcome. Obviously, the underlying probability of labels and contribution of predictor variables can be explicitly provided in LR models, which is helpful for biologists in discovering the genes that interact and cause the occurrence of disease.

The goal of classification with LR was to find a formula that gives the probability *p*_*j*_ that the *j*th sample with all its measured expressions *S*_·*j*_ represents a class 1 case. Since only two classes are considered, the probability of the sample representing class 0 is consequently 1 − *p*_*j*_. We used the following normal LR model:

(1) $$\begin{array}{c} \eta_{j}=\log\frac{p_{j}}{1-p_{j}}=\beta_{0}+\overset{M}{\underset{i=1}{\sum }}\beta_{i}x_{ij}, \end{array}$$

where *β*_0_, *β*_1_, …, *β*_*M*_ are parameters that can be estimated by maximizing the following likelihood:

(2) $$\begin{array}{c} L\left(\beta_{0},\beta \right)=\overset{N}{\underset{j=1}{\sum }}y_{j}\log p_{j}+\overset{N}{\underset{j=1}{\sum }}\left(1-y_{j}\right)\log\left(1-p_{j}\right). \end{array}$$

For microarray experiments of typical “large *p*, small *n*,” the number of samples, *N*, is usually on the order of tens, but the number of genes, *M*, is usually on the order of thousands or even tens of thousands. So the number of samples is much less than the number of variables (*N* ≪ *M*). This situation presents a number of problems when building a LR model, such as overfitting, multicollinearity of the gene expression profiles, and infinite solutions for *β*_*i*_ [17–19]. Feature selection can be used to identify the significant genes that contribute to most of the classification. Thus, some dimension-reduction techniques, such as support vector machines (SVMs), singular value decomposition, and partial least squares, are commonly used to tackle those problems and make the computation feasible [17–19]. However, the featured genes are usually selected one by one. According to the biological mechanism, genes do not work by themselves, so we employed coexpressed TSP genes in the model, as described in the following section.

### 2.2. Identification of Coexpressed Genes

Recent studies have suggested that assessing the expression of more than one gene (i.e., coexpression analysis) provides a better prediction of tumor progression than analyzing the expression of individual genes [20–22]. We identified the coexpressed gene with the paired-gene approach of the top-scoring pairs (TSP) algorithm as described by Geman et al. [16]. The TSP algorithm was originally developed for the binary classification of phenotypes according to the relative expression profiles of one-gene pair. The TSP classifier has the following advantages over the standard classifiers used in gene expression studies: (i) it is a parameter-free and data-driven machine-learning method that avoids overfitting by eliminating the need to perform specific parameter tuning, as in other machine-learning techniques, such as SVMs and neural networks; (ii) it involves only two genes, which leads to easily interpretable data and inexpensive diagnostic tests; (iii) the rank-based TSP classifiers are less affected by technical factors or normalization than classifiers which are based on expression levels of individual genes; and (iv) the simple and accurate results generated by TSP facilitate follow-up studies.

TSP gene pairs may be considered biomarker genes in a diagnostic test from microarray experiments [16, 20–22]. The methodology is being extended from one TSP gene pair to top-scoring pair of groups (TSPG) as gene signatures [20–22]. However, there are still some unresolved issues of biological explanation and the selection criteria related to the use of gene pairs instead of larger groups of significant genes. Most of the algorithms in gene selection are based on the distribution assumption of the gene expression data. However, the rank-based TSP algorithm is a parameter-free, data-driven machine-learning method. It is difficult to determine the number of gene pairs selected, but current research indicates that only a few gene pairs with the top scores need to be considered [20, 21].

For simplicity, using the gene expression matrix *D* = {*x*_*ij*_}_*M*×*N*_ with *M* genes and *N* samples, we assume that *N*_1_ samples are labeled class 0, *y*_*j*_ = 0  (*j* ∈ {1,…, *N*_1_}), and *N*_2_ samples are labeled class 1, *y*_*j*_ = 1  (*j* ∈ {*N*_1_ + 1,…, *N*}), and *N*_1_ + *N*_2_ = *N*. In this method, we focused on detecting “marker gene pairs” (*u*, *v*) because there is a significant difference in the probability of observing *G*_*u*_ < *G*_*v*_ between class 1 and class 0, where *G*_*u*_ and *G*_*v*_ denote the *u*th and *v*th rows of *D*. The conditional probabilities of observing *G*_*u*_ < *G*_*v*_ in each class are defined as

(3) $$\begin{array}{c} p_{uv}\left(0\right)=P\left(G_{u}<G_{v}\;|\;c=0\right)=\frac{1}{N_{1}}\overset{N_{1}}{\underset{j=1}{\sum }}I\left(x_{uj}<x_{vj}\right),\\ p_{uv}\left(1\right)=P\left(G_{u}<G_{v}\;|\;c=1\right)=\frac{1}{N_{2}}\overset{N}{\underset{j=N_{1}+1}{\sum }}I\left(x_{uj}<x_{vj}\right), \end{array}$$

where *I*(*x*_*uj*_ < *x*_*vj*_) is the indicator function defined as

(4) $$\begin{array}{c} I\left(x_{uj}<x_{vj}\right)=\{\begin{array}{cc} 1, & x_{uj}<x_{vj},\\ 0, & x_{uj}\geq x_{vj}, \end{array}\;j=1,2,\ldots ,N. \end{array}$$

The typical TSP method is based on maximizing the following score of (*u*, *v*) defined by German et al. [16]:

(5) $$\begin{array}{c} \Delta_{uv}=\left|p_{uv}\left(0\right)-p_{uv}\left(1\right)\right|. \end{array}$$

This approach has been shown to be as accurate as SVMs and other more sophisticated methods [20–22]. Although maximizing delta identifies the best classifier with high accuracy, it may be associated with relatively low sensitivity or specificity, as pointed out by German et al. and Ummanni et al. [16, 23]. For example, in the classification of cancer versus normal samples, accuracy is defined as the ratio between the number of correctly predicted samples and the total number of samples, and sensitivity (resp., specificity) is the ratio between the number of correctly predicted cancer (resp., normal) samples and the total number of cancer (resp., normal) samples [16]. This low sensitivity or specificity restricts us to use the classifier of one TSP for making medical decisions. This issue was improved with the use of multiple gene pairs as the classifier, which can achieve similar scores with high accuracy, sensitivity, and specificity [20–22]. Thus, we considered not just one but multiple TSP gene pairs in our models.

### 2.3. Evaluation of the Model Using Published Datasets

To evaluate the efficiency of the TSP-based LR model, we applied our model to datasets with both clinical parameters and gene expression values. We selected a dataset with a large sample size because we could obtain more reliable estimates of the efficiency of the classifiers. The dataset was from the recently published study of Sboner et al. [5], who analyzed gene expression in patients with up to 30 years of clinical follow-up data. Men who died within 10 years of being diagnosed with PCa were considered to have “lethal” disease, and those who survived at least 10 years after diagnosis were considered to have “indolent” disease. There were 165 men with lethal and 116 with indolent disease. The GS, tumor percentage, and presence of an estrogen-regulated gene (ERG) rearrangement were provided for each patient in the study. The expression of 6,100 genes was assessed using a custom gene expression array (GSE 16560).

For our model, we first randomly separated the 281 samples into a learning set with 186 samples and a validation set with the other 95 samples, with an approximately equal proportion of men with lethal and indolent PCa in each group. The learning set was utilized to create the models whose performance was evaluated in the validation set by means of the area under the receiver operating characteristic (ROC) curve (AUC). To compare the performance of our model, we performed the statistical testing based on the null hypothesis that there is no difference between the AUCs of Sboner's models and ours. Similar to the estimation of AUCs in [5], the corresponding 95% confidence intervals of the AUCs were computed in 100 iterative 10-fold cross validation procedures that enabled an unbiased estimation of the model's performance since the evaluation was performed on an independent dataset. The model is inferred to be better only if its AUC is statistically larger than that of the other models. In the original study, the authors conducted an extensive comparative analysis of the most frequently used classification methods, including the k-nearest neighbor, the nearest template prediction, diagonal linear discriminate analysis, SVMs, and neural network analysis. Their results allowed us to compare the performance of the TSP-based LR classifiers with that of the other classifiers.

To optimize and select the best models, we adopted an iterative cross validation procedure within the learning set that was similar to the procedure used by Sboner et al. [5]. The stratified tenfold cross validation procedure split the learning set into 10 disjointed partitions, test_*i*_  (*i* = 1,…, 10), with approximately equal proportions of lethal and indolent cases in each. For a given partition, test_*i*_, the models were fitted using all the other cases in the learning set, that is, the training_*i*_ set and then were evaluated with AUC analysis of test_*i*_. In the procedure of 10-fold cross validation, the model_*i*_  (*i* = 1,…, 10) was first parameterized in the training_*i*_ sets and then the corresponding AUC ontest_*i*_  (*i* = 1,…, 10) sets were calculated from model_*i*_. To avoid potential biases in the selection of the 10 partitions, the entire procedure was repeated 100 times, for 1,000 different partitions. We identified the best model with the largest AUC by comparing them as obtained in the 100 iterations. Furthermore, the featured gene pairs and estimated parameters in the model were also considered as the best model in learning set. The rationale was that the results of this procedure enable the identification of the best model, which can then be used to build a classifier that was finally evaluated on the validation set.

During the iterations of our cross validation procedure, the feature-selection procedure was carried out to identify the subsets of genes that are expressed differently in the lethal and indolent samples. In the study by Sboner et al. [5], a two-sided *t*-test was performed for each gene to identify the differently expressed genes. We then compared our models using TSP-selected coexpressed genes with the models described by Sboner et al. [5].

## 3. Results

**Figure 1:**
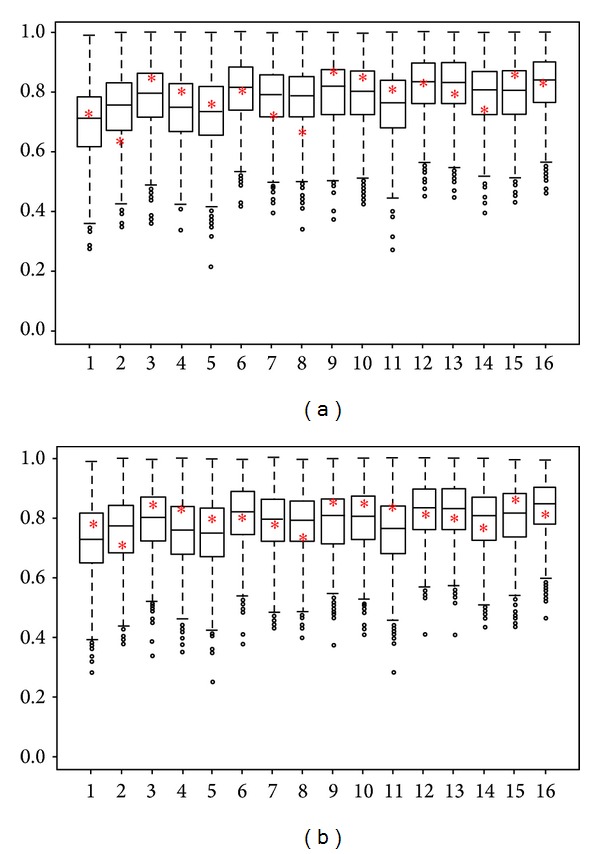
AUC boxplots for 100 tenfold cross validations of 16 models that include TSP-selected genes. The *x*-axis is the index of 16 models listed in Table 1, and the *y*-axis is the AUC values. The red star denotes the corresponding AUC values of the validation dataset that uses the best logistic regression models from the learning dataset. (a) Models that included one pair of TSP-selected genes and (b) those that included two such gene pairs.

We proposed the LR models by combining TSP-selected genes and clinical features to identify and predict the patients whose PCa will progress. The performance of the models was evaluated with dataset GSE 16560.  Table 1 lists the 16 LR models that we tested. Our models include all possible combinations of the following variables: age, GS, tumor percentage, presence of ERG gene rearrangement, and TSP-selected genes. The AUCs of 1,000 different partitions were calculated to select the best models.  Figure 1(a) shows the AUC boxplots for the 100 tenfold cross validations of the 16 models listed in Table 1, with one pair of TSP-selected genes per model. The red stars denote the AUC values from the validation datasets that correspond to the best LR models from the learning dataset.  Figure 1(b) shows the AUC boxplots for the same 16 models but with two TSP-selected gene pairs per model.

**Figure 2:**
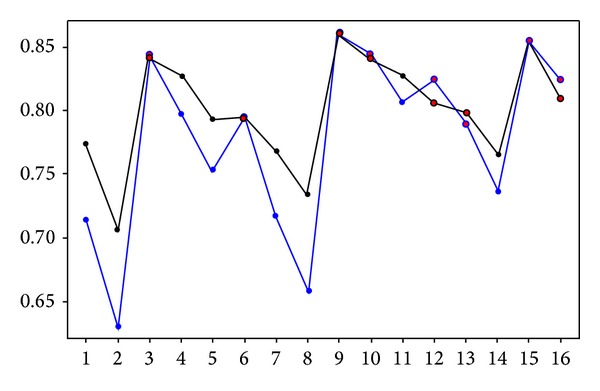
The AUCs of the 16 best models from the validation dataset. The *x*-axis is the index of the 16 models listed in Table 1, and the *y*-axis is the AUC values. The blue line shows the AUCs from the models with one TSP-selected gene pair, and the black line shows those from models with two TSP-selected gene pairs. The points circled in red are the AUCs in the 8 models that included the Gleason score as a variable.

We plotted the AUC values of the validation dataset to assess the effects of the variables on the models (Figure 2). The blue line represents AUC values from the models with one TSP-selected gene pair, and the black line represents those from the models with two TSP-selected gene pairs. Furthermore, we tested the statistical significance of the models based on the null hypothesis that there is no difference between the AUCs of Sboner's models. It is found that most of AUC values in Sboner's models were out of the 95% confidence intervals of AUCs in our models. So our models can provide an alternative in predicting prostate cancer progression. The addition of TSP-selected gene pairs can improve our models' prediction of PCa progression, which differed from Sboner's results.

What is the role of TSP-selected gene pairs in comparison with the fusion ERG and the other clinical features, especially GS? Obviously, the GS was the most statistically significant variable because all the top models included it. In Figure 2, the red circles label the 8 models that include the GS. The AUCs in those 8 models were much higher than they were in the others and were very similar in the one- and two-gene-pair models. The 8 AUCs were more than 0.8, as shown in Figure 2, so we can conclude that the models with TSP-selected gene pairs performed better than all of Sboner's models, for which the largest AUC was  0.79 in [5].

The model using only the GS yielded an AUC of 0.76; by adding fusion ERG, the largest AUC observed by Sboner et al. was 0.79 [5]. Similarly, the other models that used only GS and tumor percent (or age) without molecular profiles could yield a higher AUC if fusion ERG was added. Therefore, the addition of fusion ERG may improve the prediction capability of models that use only clinical features [5].

However, the effect of fusion ERG was a little different in our analysis. First, our models could perform better by replacing fusion ERG with TSP-selected genes. In comparison with the best model with GS and fusion ERG (AUC, 0.79) in [5], our model 1.3 with the GS and TSP-selected gene pairs performed better, with an AUC of 0.84 (95% CI = [0.81,0.88]); our best model was model 1.9, which used GS, tumor percentage, and one TSP-selected gene pair (AUC, 0.86; 95% CI = [0.79, 0.92]), but the corresponding model reported by Sboner with fusion ERG for replacement yielded an AUC of 0.75 [5]. On the other hand, the addition of fusion ERG had little or no effect on our models that included TSP-selected gene pairs. For example, the same AUC was obtained with our Model 1.3 and 1.10, with GS, fusion ERG, and TSP-selected gene pairs. Thus, TSP-selected genes seem to have a more important effect than the fusion ERG does in predicting PCa progression.

The addition of genes other than fusion ERG could not improve the prediction capability because the best models in the Sboner study [5] lacked molecular profiles. However, some improvement was observed in our study: by replacing the molecular profiles in the Sboner models with one or two TSP-selected gene pairs, our models performed better than theirs did. For example, the best model with molecular profiles in the Sboner study used GS, age, and 12 genes and yielded an AUC of 0.75, whereas our model 1.6, which used GS, age, and TSP-selected gene pairs, yielded an AUC of 0.8 (95% CI = [0.76, 0.85]). Thus, although we added fewer genes to our model, its performance was better. Moreover, the prediction capability of Sboner's models was also improved when the same number of genes was replaced with TSP-selected gene pairs, as demonstrated in Table 2. Therefore, adding the TSP-selected genes had an important effect on the performance of the original models.

Models that use only clinical features may perform better if appropriate genes, such as those selected with the TSP algorithm, are added. To explore the effect of adding genes, we compared our approach with that used in the nine models in the original study by Sboner et al. [5] that included genes. For the comparison, we selected the same number of genes for our models. However, the featured genes in the models differed each time because the 1,000 random training and testing partitions were different in the iterative cross validation procedure.

The results of our comparison were presented in Table 2. As noted, the AUCs in our models were often higher than those in the study by Sboner et al. The prognostic models for PCa can perform better if the featured genes are selected. In particular, TSP-selected genes may play an important role. First, the AUC of the model using only 18 genes increased from 0.71 in Sboner's study to 0.74 (95% CI = [0.71, 0.77]) in our model. Further, the AUCs of the models that used one and two TSP-selected gene pairs were 0.71 (95% CI = [0.67, 0.74]) and 0.77 (95% CI = [0.73, 0.81]), respectively. Thus, the TSP-based models performed better with a smaller number of genes.

The model from the Sboner study that included the GS and 16 genes did not perform any better than their model did that used the GS only, with AUCs of 0.75 and 0.75, respectively [5]. However, the AUC of our model that included the GS and 16 TSP-selected genes was 0.81 (95% CI = [0.76, 0.85]) in Table 2, and the models that used GS and one (or two) TSP gene pair(s) performed better, with an AUC of 0.84 in Figure 2.

Finally, of all the models tested in the original study, the one that included the GS and the ERG rearrangement (with no gene expression data) had the highest AUC value, 0.79 [5], whereas most of the AUC values for our models were higher than that. Therefore, in contrast to the conclusion reached by Sboner et al., we believe that adding the molecular profiles can improve the results obtained with the traditional prognostic models of PCa if the appropriate genes are selected.

From the results in Figure 2 and Table 2, we may conclude that the models' performance was not improved by the addition of large numbers of genes but was improved by the addition of significant clinical features and molecular profiles. For example, adding one TSP-selected gene pair is enough if the important clinical variables, such as GS, are included in the model. However, in the case of model 1.1, which included only one gene signature, and models 1.2 and 1.8, which also included age, the addition of more gene pairs can greatly improve the performance. Obviously, the gene selection strongly depends on the patient samples and so some statistical techniques such as bootstrap, repeated sampling, or cross validation were commonly used in the TSP-extended algorithms. In the current research, the computation cost of TSP-based algorithms is not the main concern, but the topics about the optimal number of gene pairs added to improve the clinical models are still interesting in further research.

## 4. Conclusion and Discussion

We have introduced an LR-based classification method that combines TSP-selected genes and clinical measurements. The empirical results of [19, 20] based on the datasets of prostate cancer progression show that the classification models using one or two TSP-selected gene pairs perform better than most commonly used one-gene-at-a-time approaches. With the combination of LR, our models not only preserved the basic advantages of the TSP algorithm but also incorporated the clinical features. Furthermore, the LR-TSP model provides the underlying probability of predictionand coexpressed genes that are used as biomarkers in the model. Thus, our proposed method provides explicit biologic interpretation of the clinical tests. Based on the statistical inference with the iterative cross validation, the better performance was shown in our models.

As mentioned in the report of Sboner et al. [5], many factors can influence the performance of the models, such as the definitions of lethal and indolent PCa, the use of samples contaminated with stromal tissue, the selection of genes assayed using a DASL (cDNA-mediated annealing, selection, extension, and ligation) array, and the effect of intertumor heterogeneity. Based on the study of GSE 16560, we explored the possible effect of genes used in the clinical models. The featured genes are often selected by using a one-gene-at-a-time approach. Sboner et al. performed a two-sided *t*-test for each gene within the training_*i*_ partition, thereby avoiding overfitting because the selection of the genes was performed on only training sets [5]. They also implemented stepwise-like feature selection, sorted the genes according to their *P* values from the *t*-testing, and then added the genes to their models. Our study, on the other hand, demonstrates that coexpression analysis yields better prediction of tumor progression than the analysis of individual genes does. Therefore, we conclude that TSP selection is a useful tool for feature (and/or gene) selection to use in prognostic models.

## Figures and Tables

**Table 1:** Logistic regression models that included TSP-selected gene pairs and different combinations of clinical variables.

Model number	Patient's age	Gleason score	Tumor percentage	Fusion ERG arrangement	TSP genes
1.1					X
1.2	X				X
1.3		X			X
1.4			X		X
1.5				X	X
1.6	X	X			X
1.7	X		X		X
1.8	X			X	X
1.9		X	X		X
1.10		X		X	X
1.11			X	X	X
1.12	X	X	X		X
1.13	X	X		X	X
1.14	X		X	X	X
1.15		X	X	X	X
1.16	X	X	X	X	X

**Table 2:** Comparison of the performance of our logistic regression models with that of the nine models evaluated by Sboner et al. [5], using the same number of genes.

Model number	Patient's age	Gleason score	Tumor percentage	Fusion ERG	Number of genes	AUC in ref. [5]	AUC of our model
2.1				X	18	0.672	0.769
2.2	X			X	9	0.708	0.732
2.3					18	0.713	0.736
2.4		X		X	21	0.726	0.793
2.5	X				11	0.730	0.712
2.6	X	X	X		3	0.738	0.806
2.7	X	X		X	12	0.745	0.804
2.8		X			16	0.749	0.813
2.9	X	X			12	0.750	0.788
